# STCP-Net: A few-shot learning approach based on spectral-temporal convolution and an improved prototypical network for ECG classification

**DOI:** 10.1371/journal.pone.0357954

**Published:** 2026-10-15

**Authors:** Jia Li, Dainan Gan, Xiaolin Zhan, Wei Yao, Bin Zou, Bin Zhang, Dong Li, Dan Li, Lin Li

**Affiliations:** 1 School of Electronics and Information Engineering, Wuyi University, Jiangmen, China; 2 School of Mathematics and Computational Science, Wuyi University, Jiangmen, China; 3 Department of Cardiovascular Disease and Clinical Experimental Center, Jiangmen Central Hospital, Jiangmen, China; 4 Department of Intensive Care Unit, Jiangmen Central Hospital, Jiangmen, China; 5 Department of Electrocardiogram, Jiangmen Central Hospital, Jiangmen, China; 6 Department of Rehabilitation, Shengjing Hospital of China Medical University, Shenyang, China; Polytechnic University of Marche: Universita Politecnica delle Marche, ITALY

## Abstract

To address the heavy reliance of deep learning models on manually labeled data in ECG classification, we need to tackle two major limitations. First, sample sizes are often limited in clinical settings, making data annotation costly and impractical. Second, existing few-shot learning methods still fall short in two key areas: 1) Existing strategies pay insufficient attention to rare arrhythmia types. This makes it difficult to effectively identify rare clinical cases. 2) Single feature representation fails to fully integrate the global frequency, local time-frequency, and semantic information of ECG signals. This results in incomplete feature representation. To overcome these challenges, we propose a novel few-shot learning method for ECG classification. In terms of feature extraction, a spectral-temporal convolution feature extractor is designed to precisely focus on the key information of ECG by efficiently integrating the global frequency, local time-frequency features and semantic information of the signal. Furthermore, with regard to classification, an improved prototypical network based on cross-class bias diminishing is developed to enhance the recognition performance for rare arrhythmia type samples. The experimental results show that the proposed method achieves significant classification performance across multiple public datasets, especially on Chapman, it attains an average accuracy exceeding 90% in the 2-way 1-shot and 2-way 5-shot tasks.

## Introduction

Cardiovascular diseases now pose a major threat to human health worldwide, as they can not only cause serious health problems but also can be fatal [1]. In clinical diagnosis, the ECG, which records the electrical activity of the heart, plays an irreplaceable role in the diagnosis and monitoring of arrhythmia [2]. However, traditional ECG analysis relies heavily on physician expertise, introducing subjectivity and potential errors. The rapid development of machine learning, particularly deep learning, has brought significant innovations to the field of arrhythmia detection. In recent years, methods such as support vector machine [3], random forest [4], neural network [5,6], AdaBoost classifier [7] have been widely applied in this field. Deep learning technology simplifies the process of manually designing features and significantly advances the progress of arrhythmia detection technology through its powerful automatic feature extraction capabilities. Representative methods include CNN [8,9], RNN [10,11], and LSTM [12,13]. However, Deep learning relies on large-scale training data, making it unsuitable for real clinical scenarios where labeled data are often insufficient due to the scarcity of rare case samples and the limitation of medical knowledge.

In order to solve the problem of insufficient clinical annotation data, researchers have begun to explore few-shot learning (FSL) [14], in which transfer learning and metric learning have become two mainstream strategies to solve the problem of ECG classification with few samples. Although encoder-decoder or U-Net style architectures have been successfully applied to biomedical modeling, for example, in hemodynamic property estimation [15] or morphological stress prediction [16], these frameworks are mainly designed for intensive prediction tasks. In contrast, our goal is to classify and discriminate in the case of extreme label scarcity, which is more naturally aligned with a few-shot classification paradigm, which learns discriminative embeddings rather than predicting continuous-valued outputs. Transfer learning can effectively alleviate the problem of insufficient medical data labels by using pre-trained models on large-scale data sets. For example, Bhaskarpandit et al. [17] adopted the transfer learning method, applying a variety of basic models pre-trained on ImageNet dataset (such as EfficientNet, VGG16, ResNet101) as feature extractors to transfer them to ECG images collected by medical institutions in Pakistan to implement few-shot classification tasks. In a 3 way-5 shot task, EfficientNet showed excellent performance with an accuracy of 96.07%. Similarly, Liu et al. [18] used EfficientNet as a feature extractor, pre-trained it on PTB dataset, and reduced redundant information through reshaping the feature dimension. Subsequently, they conducted few-shot classification experiments on MIT-BIH dataset. They achieved 93.80% accuracy in a 4way-5shot task. Metric learning does not need to rely on direct transfer model parameters, but it achieves rapid generalization of new categories by learning the similarity measurement relationship between samples. In practice, Gupta et al. [19] proposed an ECG classification method based on a siamese network, which is pre-trained on multiple data sets through similarity learning to achieve classification with only a small amount of labeled data. The experimental results show that the accuracy is improved to 92.25% under a 5way-5shot task. Likewise, Cao et al. [20] proposed RSTA-ProtoNet, an optimized prototypical network. By constructing a spatio-temporal attention residual network, it effectively extracts ECG features for few-shot classification. They achieved 91.25% accuracy in a 2way-5shot task on Chapman dataset.

In summary, at present, the application of FSL in the field of ECG classification is relatively limited and faces the following challenges: 1) The existing research mainly focuses on the classification of common arrhythmia types, demonstrating poor performance in recognizing rare arrhythmia types [21]. 2) Most existing methods rely on a single feature representation, exhibiting difficulty in extracting both global trends and local abrupt changes in ECG signals with frequency aliasing [22], thereby limiting classification performance. In response to these challenges, the main contributions of this paper are as follows:

1) We propose and validate a novel FSL method for ECG classification, named STCP-Net. The framework consists of a spectral-temporal convolution feature extractor and an improved prototypical network to effectively identify rare arrhythmia types with rare clinical data.
2) A spectral-temporal convolution feature extractor has been designed, which effectively captures multi-scale information in complex signals by fusing global spectrum features and local time-frequency features. Moreover, a semantic-boosted spatiotemporal processor efficiently extracts the spatiotemporal and semantic information of the signal, enhancing the identification of rare arrhythmia types.
3) In order to reduce the dependence of the model on a large amount of labeled data, an improved prototypical network is designed as a classifier. By introducing a cross-class bias diminishing the network can reduce the distribution difference between the support set and the query set to improve the generalization ability of new categorys.
4) We verified STCP-Net on several popular public datasets and obtained 90.51% accuracy in a 2-way 5-shot task on ECG200, and 78.35% accuracy in a 4-way 5-shot task of rare arrhythmia type samples on Chapman. The aforementioned results significantly outperform existing methods.

The remainder of this paper is structured as follows: Related work introduces related work; Methods elaborates on the technical details of the proposed method; Research design describes the experimental design; Results and analysis presents experimental results and corresponding analysis; Discussion discusses the research findings; finally, Conclusion provides a comprehensive summary of the paper.

## Related work

### DL-based ECG classification

In recent years, deep learning (DL) techniques have been continuously advanced in the field of ECG classification, providing novel perspectives and approaches for the automatic diagnosis of cardiovascular diseases and driving rapid progress in this area. For example, Zhang et al. [23] proposed SE-ECGNet, a framework that extracts multi-scale ECG features through the parallel integration of 2D and 1D convolutional blocks while employing a squeeze-and-excitation (SE) module to enhance feature representation. Similarly, Ji et al. [24] designed GruNet, which combines CNN with a gated recurrent unit and adopts an innovative encoder-decoder framework to achieve efficient extraction and fusion of multi-scale features.

However, the above methods still face challenges in effectively capturing the local features in ECG signals. In order to solve this problem, some studies have introduced signal processing techniques to better capture multi-scale information of signals. For instance, Wang et al. [22] integrated the continuous wavelet transform (CWT) with CNN, CWT was first used to map ECG signals into time-frequency two-dimensional spectra, and then CNN automatically extracted features for classification. Based on this idea, Shoughi et al. [25] proposed an ECG classification model that combines the discrete wavelet transform with CNN. Although these methods perform well in feature extraction, they depend heavily on large amounts of labeled data, making it difficult to adapt to the actual situation of data scarcity.

### FSL-based ECG classification

Metric-based FSL uses a distance as the evaluation criterion and relies on only a few labeled samples to construct the support set, which enables prediction of the query set through similarity measure in the feature space. This approach has been widely applied in image classification, target detection, bearing fault diagnosis and other fields [26]. In recent years, with the increasing complexity and scarcity of ECG data, the application of FSL technology to ECG classification has also gained growing attention. In this context, Li et al. [27] introduced a one-dimensional siamese network for arrhythmia detection, making the first exploration of FSL in the field of ECG classification. Subsequently, Liu et al. [28] further developed a meta siamese network that combines deep convolutional networks and residual networks for more effective feature extraction. The accuracy rate reached 97.72% under a 5way-5shot task. Although the siamese network has made a breakthrough in arrhythmia detection, its pairwise comparison mechanism is highly sensitive to data distribution and struggles to deal with category imbalance.

To address these issues, research has gradually shifted toward prototypical networks, which can more stably and reliably manage category diversity by capturing the overall characteristics of each category. For example, Li et al. [29] proposed a parallel multi-scale convolution based prototypical network (PM-CNN), which transfers one-dimensional ECG signals into three-dimensional RGB images and extracts their rich features through convolutional neural networks. They achieved an accuracy of 87.50% in a 2way-5shot task. To further mitigate noise interference, Yang et al. [30] developed a spectral propagation graph network (SPGN), which extends time series into the frequency domain and performs spectral comparison to better capture the spectral relationship. In a 2way-5shot task on ECGFiveDays dataset, they achieved an accuracy of 98.70%. Building upon these ideas, we explore a new strategy for obtaining ECG multi-scale information by integrating both local and global time-frequency features.

## Methods

We propose a FSL approach based on spectral-temporal convolution and an improved prototypical network (STCP-Net). Its classification process is illustrated in Fig 1(a). In the feature extraction stage, a spectral-temporal convolution feature extractor (ST-CFE), which integrates a wavelet-fourier transform block (WFTBlock)

**Fig 1:**
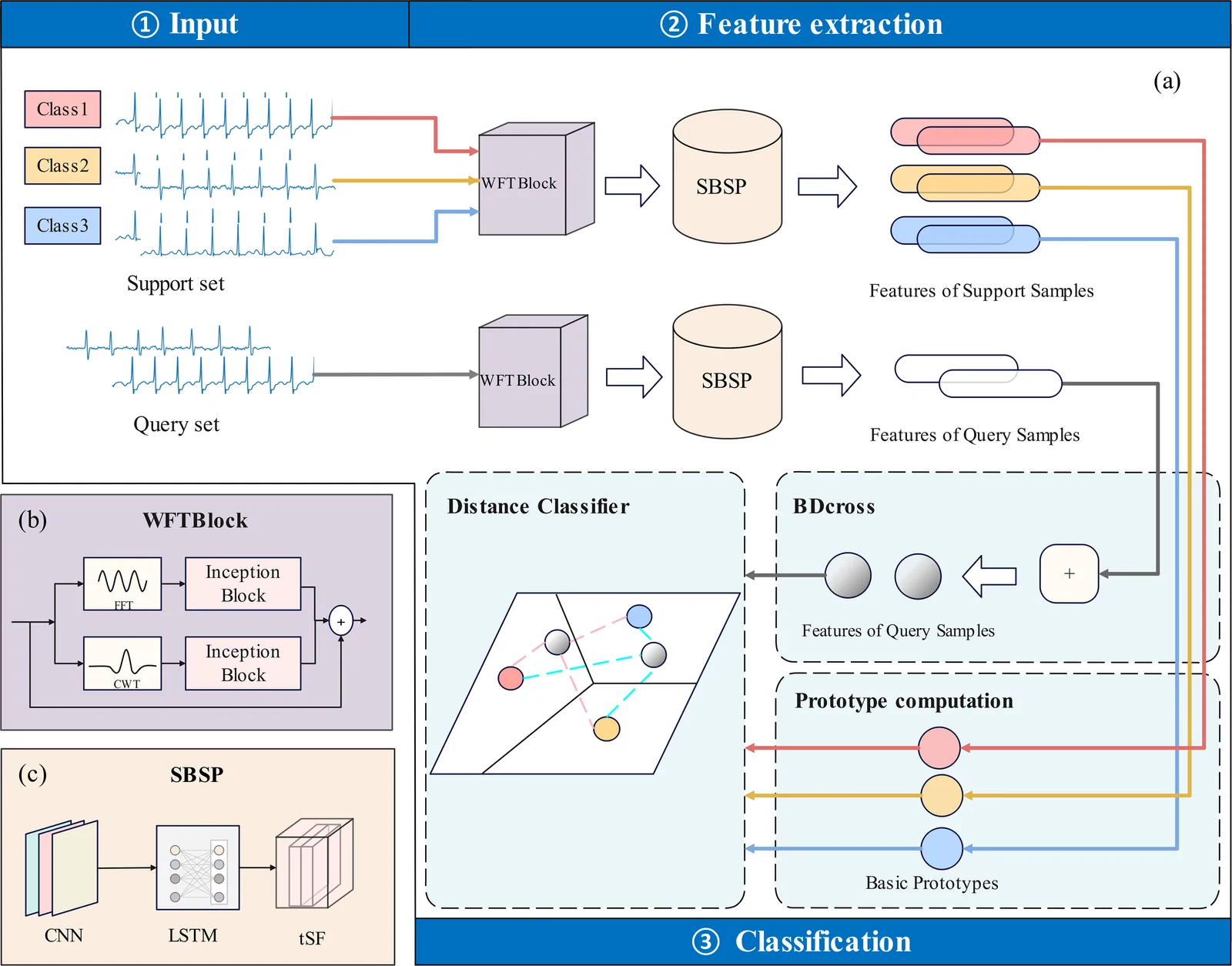
Architecture of STCP-Net. (a) Flow chart of classification. (b) WFTBlock. (c) SBSP.

(see Fig 1(b)) and a semantic-boosted spatiotemporal processor (SBSP) (see Fig 1(c)), is employed to obtain the key information of ECG signals. In the classification stage, an improved prototypical network is designed to classify the query set data by introducing cross-class bias diminishing (BDcross), which helps reduce inter-class bias and enhances classification robustness.

### ST-CFE

The WFTBlock in ST-CFE captures both global and local features of ECG signals from multiple perspectives, thus enriching the overall feature expression representation. The SBSP further enables the model to utilize global semantic information more effectively to enhance feature learning in a FSL scenario.

#### WFTBlock.

The structure of WFTBlock [31] is illustrated in Fig 2. First, the input signals $x_{t},t\in [0,T]$ are processed into the fast fourier transform (FFT) and the CWT to generate two-dimensional frequency domain images and two-dimensional time-frequency domain images, respectively. These representations contain both global and local information of the signals. Next, the resulting features are fed into the Inception module, where multi-scale convolution operation is used to further extract the deep features. Finally, by connecting it with the residual of the original input signal, the global-local features of the signal are obtained. The corresponding algorithm is shown in Algorithm 1.

**Fig 2:**
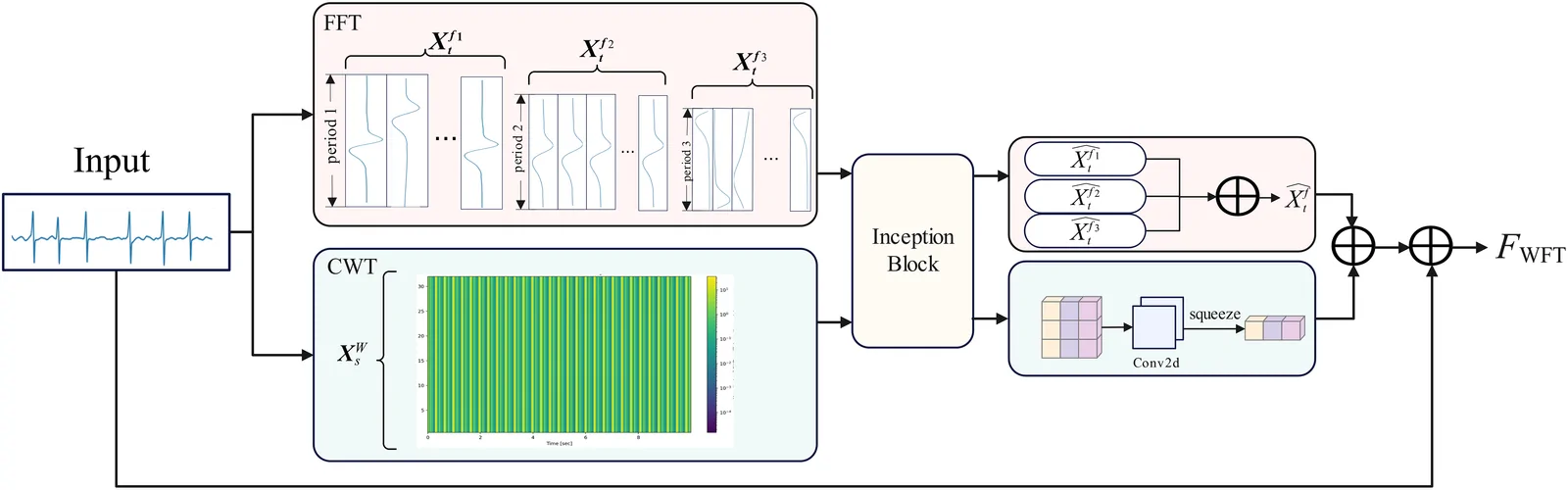
Structure of WFTBLock. *F*_WFT_:The output features of the WFTBlock. FFT: Fast fourier transform. CWT: Continuous wavelet transform.

Based on the FFT results of the input signal, the first k frequency components with the highest amplitude are selected to construct the corresponding two-dimensional frequency domain image. At the same time, CWT generates a two-dimensional time-frequency domain image by analyzing signals at different scales and translations.

**Algorithm 1. WFTBlock**



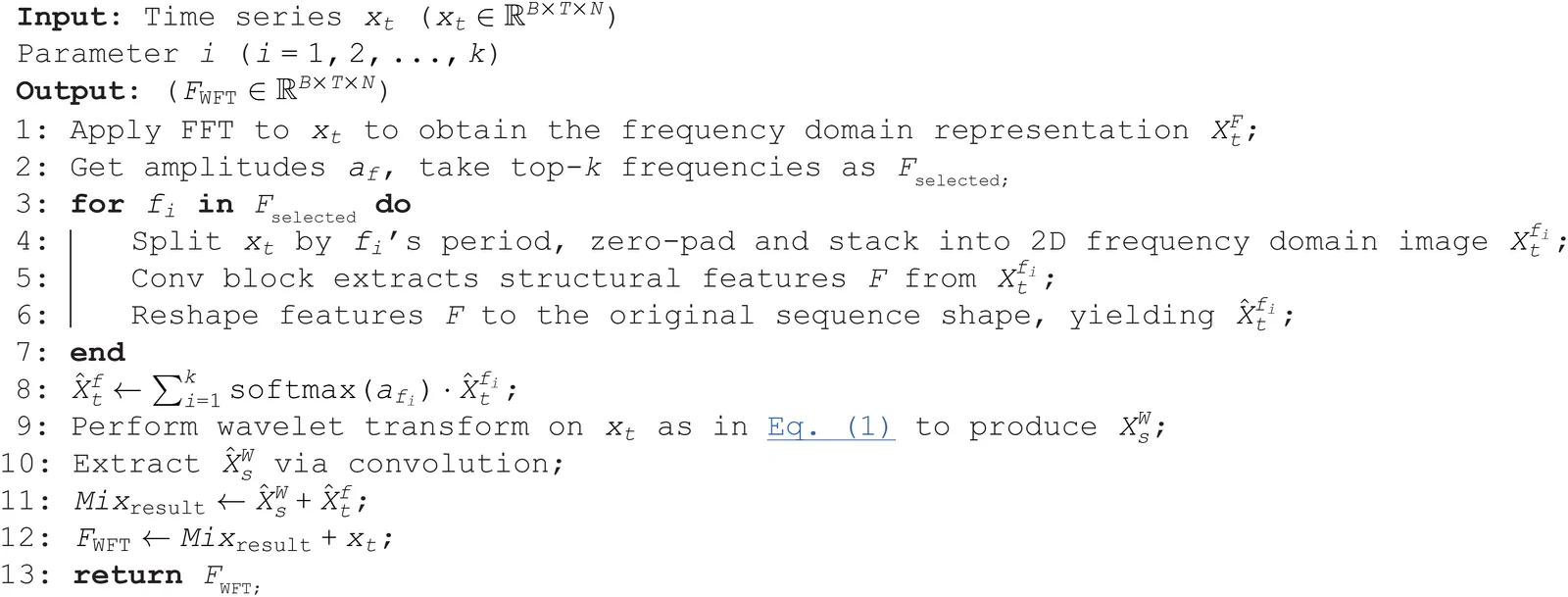



The process is expressed in Eq (1):


$$\begin{array}{c} CWT\left(s,r\right)=\int x_{t}\cdot \frac{1}{\sqrt{s}}\cdot \psi^{*}\left(\frac{t-r}{s}\right)dt \end{array}$$ (1)

Here, *s* denotes the wavelet scale, *r* represents the translation factor, ψ is the Sym 4 wavelet function, and * indicates the complex conjugate operation. After that, the results of CWT and FFT are fed into the Inception module, where deep features are extracted by using convolution kernels of various scales. For the CWT output, multi-dimensional features are compressed into one-dimensional feature vectors through convolution kernels. For the FFT output, the data is reshaped to match the original sequence and combined with the amplitude weighting of each frequency component. Finally, the features extracted from FFT and CWT are fused, and a residual connection is introduced after the WFTBlock to ensure that the original input information is fully preserved.

#### SBSP.

The structure of SBSP, illustrated in Fig 3, consists of multiple channel-varying one-dimensional convolution blocks, a long short-term memory network (LSTM) and a temporal semantic filter (tSF). First, the one-dimensional convolution blocks extract the local features from the input data. Next, the LSTM module captures the long-term dependencies and enriches the time series information. Finally, the tSF module is introduced to enhance the global semantic understanding of the data.

**Fig 3:**
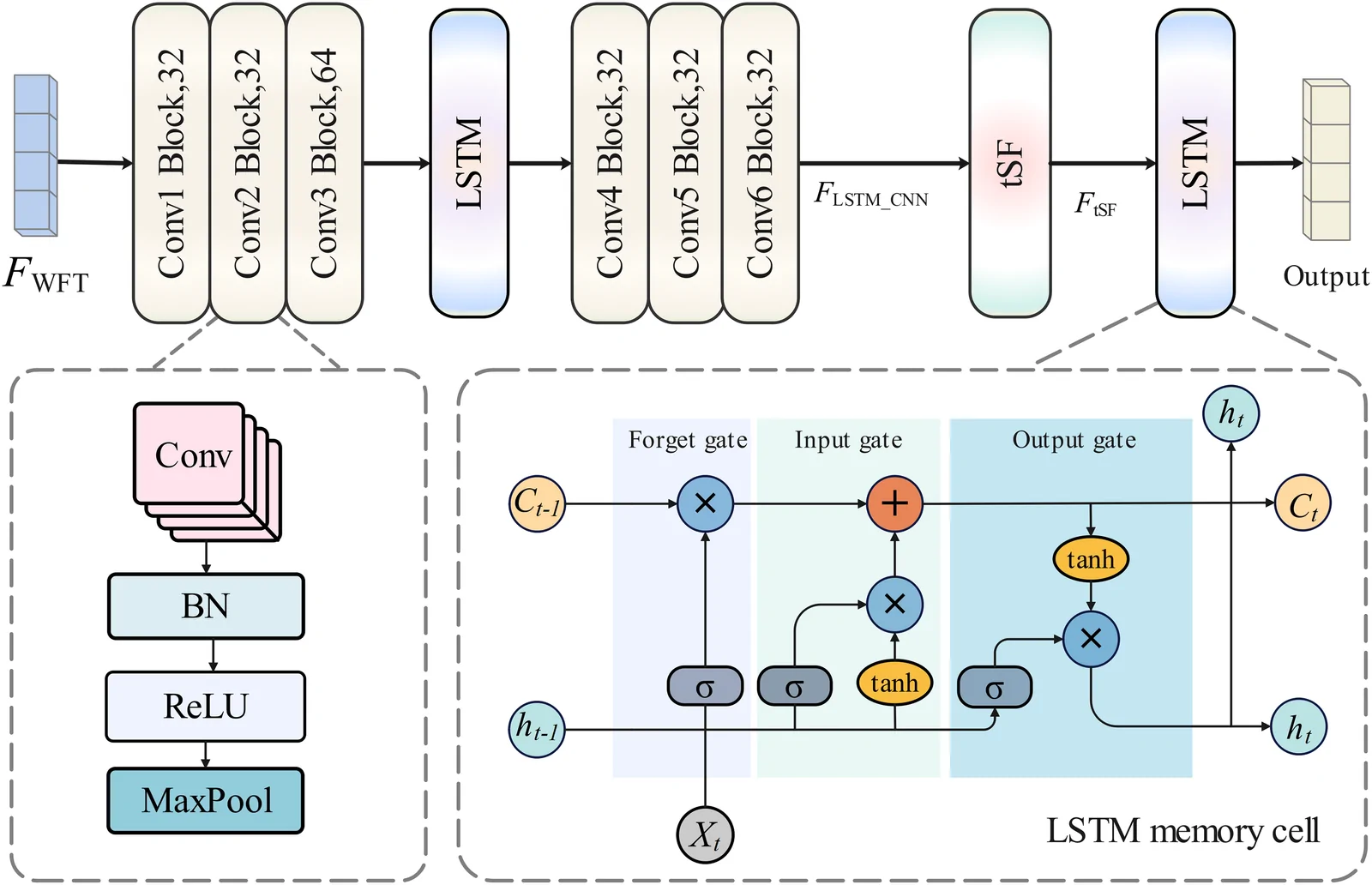
Structure of SBSP. $F_{\text{LSTM\_CNN}}$: The output features of the Conv6 Block. *F*_tSF_: The output features of the tSF. *F*_WFT_: The output features of the WFTBlock. BN: Batch normalization. Conv: Convolution.

CNN uses six one-dimensional convolution blocks to extract the spatial features of the signal. Each convolutional block contains a one-dimensional convolutional layer, followed by a batch normalization, a ReLU activation function, and max pooling. All convolutional layers use 1×3 convolution kernels. The number of channels in the first three layers is progressively increased to enhance feature expression, while the last three layers are reduced to compress redundant information.

The LSTM [32] efficiently learns long-term dependencies by introducing the cell state and three gating mechanisms. At each time step t, the LSTM receives the current input $x_{t}$, the hidden state of the previous moment $h_{t-1}$, and the previous cell state $c_{t-1}$. First, the forget gate computes a gating signal $g_{f}(t)$ that determines which information should be discarded from the cell state $c_{t-1}$, as shown in Eq (2). Next, the input gate calculates a gating signal $g_{i}(t)$ to decide how much new information should be added to the new cell state $c_{t}$, and generates a candidate cell state ${\tilde{c}}_{t}$, as shown in Eqs (3) and (4). Then, the LSTM combines the results of the forget gate and the input gate to update the new cell state $c_{t}$, as given in Eq (5). Finally, the output gate computes a gating signal $g_{O}(t)$ that decides which part of $c_{t}$ will be output at the current time step, producing the new hidden state $h_{t}$, as shown in Eqs (6) and (7).


$$\begin{array}{c} g_{f}(t)=\sigma \left(w_{f}\left[h_{t-1},x_{t}\right]+b_{f}\right) \end{array}$$ (2)

$$\begin{array}{c} g_{i}(t)=\sigma \left(w_{i}\left[h_{t-1},x_{t}\right]+b_{i}\right) \end{array}$$ (3)

$$\begin{array}{c} {\tilde{c}}_{i}(t)=tanh\left(w_{c}\left[h_{t-1},x_{t}\right]+b_{c}\right) \end{array}$$ (4)

$$\begin{array}{c} c_{t}=g_{f}(t)⊙c_{\text{t-1}}+g_{i}(t)⊙\tilde{c}(t) \end{array}$$ (5)

$$\begin{array}{c} g_{o}(t)=\sigma \left(w_{o}\left[h_{t-1},x_{t}\right]+b_{o}\right) \end{array}$$ (6)

$$\begin{array}{c} h_{t}=g_{o}(t)⊙tanh(c_{t}) \end{array}$$ (7)

Here, σ denotes the sigmoid activation function, *tanh* represents the hyperbolic tangent activation function. $w_{f}$, $w_{i}$, $w_{c}$, $w_{o}$ denote the corresponding weight matrix, while $b_{f}$, $b_{i}$, $b_{c}$, $b_{o}$ represent the bias vector. The ⊙ symbol indicates element-wise multiplication.

The structure of tSF [33] is illustrated in Fig 4. In this module, the query (Q) is generated by the input features, while the keys (K) and values (V) are derived from the learnable parameters θ. First, the model produces a set of learnable parameters as K and V. By calculating the correlation between Q and K, an attention weight matrix is obtained. Next, the context vector is generated by using the calculated attention weight and the learnable V dot product. Then, the residual connection is performed with the original input features to integrate the global semantic information into the original input features. Finally, the enhanced features are sent to the feedforward network (FFN) for further optimization to generate the final output features. This process is based on the interaction of features, enabling information exchange between a single input sample and the entire dataset.

**Fig 4:**
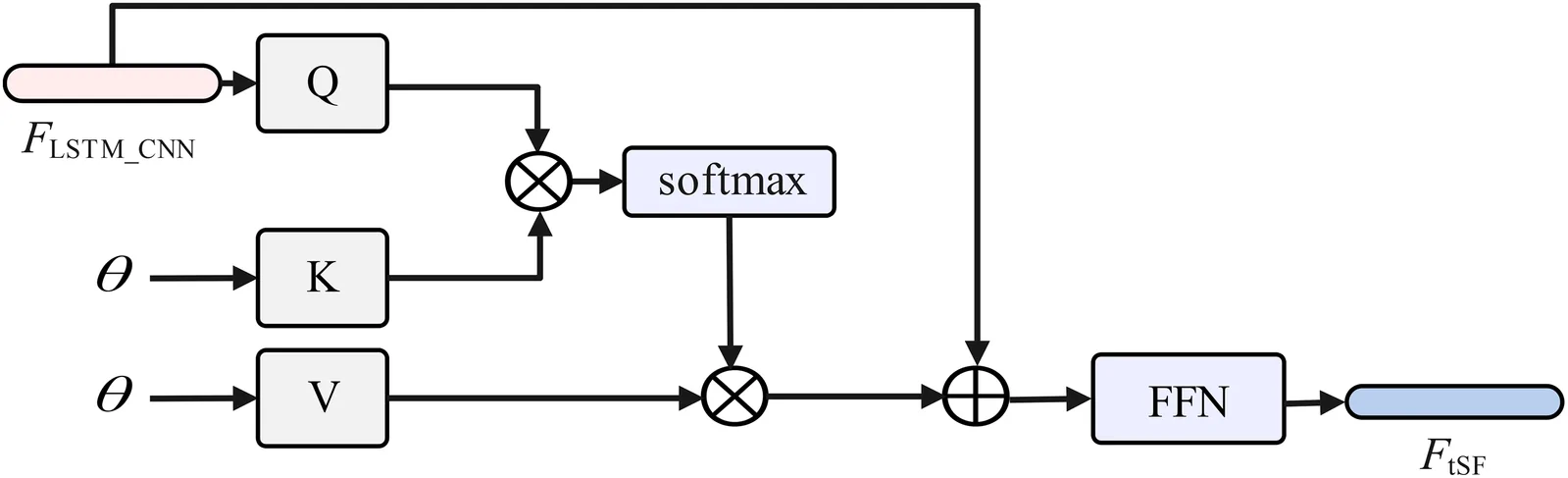
Structure of tSF. $F_{\text{LSTM\_CNN}}$: The output features of the Conv6 Block. *F*_tSF_: The output features of the tSF. FFN: Feedforward network.

### Improved prototypical network

By introducing BDcross [34] into the existing prototypical networks [35], we propose an improved prototypical network for ECG classification. Firstly, the mean value of the category features of the support set samples is calculated to obtain various prototype vectors. BDcross is then used to adjust the features of the query set samples. Finally, the recognition of the query set samples is achieved by calculating the distance between the features of the query set samples and the prototype for each category.

Specifically, the improved prototypical network first utilizes the features of the support set samples to calculate the prototype of each category, which serves as the representative vector of the category. For each category, the prototype $p_{c}$ is defined as:


$$\begin{array}{c} p_{c}=\frac{1}{\left|S_{c}\right|}\sum_{\left(x_{s,i},y_{s,i}\right)}f_{\theta }(x_{s,i}) \end{array}$$ (8)

Here, $x_{s,i}$ denotes a support set sample, $f_{\theta }(x_{s,i})$ represents the embedding vector of $x_{s,i}$ mapped to the ST-CFE, and $\left|S_{c}\right|$ indicates the number of samples in category *c* within the support set. Then, BDcross is used to calculate the mean difference between the support set and the query set in the feature space, yielding the distribution deviation. The deviation is subsequently added to the features of the query set samples to reduce the distribution difference between the two domains. This process can be expressed as:


$$\begin{array}{c} \xi =\left|\frac{1}{S}\right|\cdot \sum_{i=1}^{\left|S\right|}f_{\theta }(x_{\text{s,i}})-\left|\frac{1}{Q}\right|\cdot \sum_{j=1}^{\left|Q\right|}f_{\theta }(x_{\text{q,j}}) \end{array}$$ (9)

$$\begin{array}{c} f_{\theta }^{\prime }(x_{\text{q,j}})=f_{\theta }(x_{\text{q,j}})+\xi \end{array}$$ (10)

Among them, ξ denotes the deviation, |*S*| represents the number of samples of the support set, |*Q*| represents the number of samples of the query set. $f_{\theta }(x_{\text{s,i}})$ denotes the features of the support set samples, $f_{\theta }(x_{\text{q,j}})$ denotes the features of the query set samples, and $f_{\theta }^{\prime }(x_{\text{q,j}})$ represents the features of the query set samples after the BDcross adjustment. Finally, the Euclidean distance between the query sample features and the prototypes is calculated. The distances are converted into prediction probabilities using the softmax function, and the model parameters are optimized with cross-entropy loss. The prediction probability of the query set samples belonging to category *c* can be expressed as:


$$\begin{array}{c} p_{\theta }(y_{q,j}=c|x_{q,j})=softmax(-d(f_{\theta }^{\prime }(x_{q,j}),p_{c}))=\frac{\exp(-d(f_{\theta }^{\prime }(x_{q,j}),p_{c}))}{\sum_{c=1}^{N}\exp(-d(f_{\theta }^{\prime }(x_{q,j}),p_{c}))} \end{array}$$ (11)

Here, $p_{\theta }$ denotes the prediction probability and d(·) represents the selected euclidean distance function.

## Research design

### Experimental data

Chapman is supported by the support of Chapman University and Shaoxing People ’s Hospital for arrhythmia research and includes 12-lead electrocardiogram data [36]. This database contains ECG data from 10,646 patients with a sampling rate of 500 Hz, covering 11 different arrhythmia categories. The common categories include atrial premature beat (APB), atrial fibrillation (AFIB), sinus arrhythmia (SI), sinus bradycardia (SB), sinus rhythm (SR), sinus tachycardia (ST) and supraventricular tachycardia (SVT), totaling 7 categories. The rare categories include atrial tachycardia (AT), atrioventricular nodal reentrant tachycardia (AVNRT), atrioventricular reentrant tachycardia (AVRT) and sinus atrial wandering rhythm (SAAWR), totaling 4 categories, as shown in Table 1. In this study, incomplete records were filtered out, and lead II was selected as the input for the model.

**Table 1:** Number of categories in Chapman.

Class	SB	SR	AFIB	ST	AF	SI	SVT	AT	AVNRT	AVRT	SAAWR
Numbers	3888	1826	1780	1568	445	399	587	121	16	8	7

The UCR archive is an important open-source dataset resource in the field of time series mining [37] and is widely used in research on time series classification tasks. In this study, the ECG200 and ECG5000 were used, with detailed information provided in Table 2. All data sets (Chapman, ECG200, ECG5000) are cleansed data provided by the original publisher. Because the data sets themselves contain high-quality preprocessing, this study does not need to perform additional filtering or segmentation, and only performs normalization processing.

**Table 2:** Details of sets in the UCR archive.

Sets	Size	Length	No. of Classes	Classes
ECG200	200	96	2	MI, N
ECG5000	5000	140	5	SP or EB, R on -T, PVC, UB, N

As an important open-source dataset resource in the field of time series mining, the UCR archive is widely used in the research of time series classification tasks [37]. The ECG200 and ECG5000 are employed with detailed information is shown in Table 2. In the few-shot experiment, we split the dataset $D=\left(x_{1},y_{1}),(x_{2},y_{2}),......,(x_{n},y_{n}\right)$ is split into a meta-training set $D_{train}$ and a meta-testing set $D_{test}$ and the ’ N-Way K-Shot ’ method from meta-learning is used to train the model. For the Chapman, seven common categories are used to construct the meta-training set, while four rare categories form the meta-testing set. For the ECG200 and ECG5000, the original training and test sets provided in the UCR archive are used as the meta-training and the meta-test set, respectively. The specific distribution of the datasets is shown in Table 3.

**Table 3:** Number of ECGs in meta-training set and meta-testing set.

Sets	Training	Testing	Total
ECG200	100	100	200
ECG5000	500	4500	5000
Chapman	10493	152	10645

### Implementation details

The proposed method is developed based on the PyTorch framework and runs on an NVIDIA Tesla T4 GPU. For model optimization, we adopt the Adam optimizer with an initial learning rate of 0.001. To enhance network performance, the learning rate is reduced by half every 40 epochs. During the meta-training phase of the improved prototypical network, 200 few-shot tasks are randomly sampled from the meta-training set in each epoch to update the model. In the meta-testing phase, 100 tasks are drawn from the meta-test set per epoch for evaluation. The final performance is reported with a 95% confidence interval based on statistical analysis. The specific parameters are shown in Table 4.

**Table 4:** Hyperparameters of STCP-Net.

Parameters	Value
epoch	50
lr	0.001(1−40epoch),0.0005(41−50epoch)
optimizer	Adam
meta-training tasks	200
meta-testing tasks	100

### Evaluation indicators

We use accuracy (Acc) (Eq (12)), precision (Eq (13)), recall (Eq (14)) and F1 (Eq (15)) as evaluation indicators. Acc represents the proportion of the number of correctly predicted samples to the total number of test samples. Recall rate measures the ability of the model to identify the true positive class. Precision reflects the reliability of the predicted positive results. F1 comprehensively evaluates the performance of the model by the harmonic mean of the precision rate and the recall rate. Here, TP, TN, FP, and FN denote true positives, true negatives, false positives, and false negatives, respectively.


$$\begin{array}{c} Acc=\frac{TP+TN}{TP+TN+FP+FN} \end{array}$$ (12)

$$\begin{array}{c} precision=\frac{TP}{TP+FP} \end{array}$$ (13)

$$\begin{array}{c} recall=\frac{TP}{TP+FN} \end{array}$$ (14)

$$\begin{array}{c} F1=\frac{2TP}{2TP+FP+FN} \end{array}$$ (15)

## Results and analysis

### Comparison study

The comparison results on Chapman are presented in Table 5. Compared with CAO, our method shows superior performance across various settings, for instance, achieving 90.63% accuracy in the 2-way 1-shot scenario, which exceeds CAO by about 7 percentage points. In addition, compared with the mainstream FSL methods such as MatchingNet and PrototypicalNet, our method also performs well. The above results show that our method has significant advantages in the few-shot learning scenario. To evaluate the overall statistical significance across multiple methods, we perform a one-way ANOVA on the accuracy results of 5 independent runs for each few-shot task. The resulting p-values are all less than 0.001, indicating that at least one method differs significantly from the others.

**Table 5:** Few-shot classification accuracy (%) on Chapman. Bold numbers indicate the best performance in each column. (mean±error range).

Methods	Acc (4-way, 1-shot)	Acc (4-way, 5-shot)	Acc (2-way, 1-shot)	Acc (2-way, 5-shot)
MatchingNet [38]	51.44±1.30	55.34±1.25	78.10±0.95	82.33±0.63
PrototypicalNet [35]	50.97±1.57	66.49±1.19	75.10±1.38	88.05±0.77
CAO [20]	63.42±0.15	73.75±0.11	83.48±0.16	91.25±0.14
STCP-Net (Ours)	**66.06±2.64**	**78.35±1.23**	**90.63±0.56**	**93.73±0.64**

The comparison results on the set in the UCR file are illustrated in Table 6. Our approach is superior to the existing methods in most indicators. For example, on ECG200, our method has an accuracy rate of 90.51%, which is 9.81% higher than YANG ’s 80.7%. On ECG5000, our method has an accuracy of 63.06%, which is slightly higher than YANG ’s 61.4%.

**Table 6:** Few-shot classification accuracy (%) on ECG200 and ECG5000. Bold numbers indicate the best performance in each column. (mean±error range).

Methods	Acc (2-way, 5-shot, ECG200)	Acc (5-way, 5-shot, ECG5000)
YANG [30]	80.70±0.33	61.40±0.19
STCP-Net (Ours)	**90.51±0.66**	**63.06±0.91**

### Ablation study

In this paper, CNN-LSTM and prototypical network are used as baseline models, and systematic ablation experiments are conducted on Chapman. The experimental results are shown in Table 7. Compared with the baseline model, the introduction of the tSF module improves the accuracy rate across all settings. Especially in the 4-way 5-shot task, the accuracy rate increases by 3% to 75.93%, indicating that the tSF module effectively extracts global semantic information. Furthermore, introducing the WFTBlock module enhances the model representation capability. In particular, in the 4-way 5-shot task, the accuracy rate reaches 78.31%, which is more than 5% higher than the baseline, which proves that the WFTBlock module can effectively extract the key information in the signal. Finally, after the introduction of BDcross, the performance under all experimental settings is significantly improved, which indicates that BDcross is particularly critical for sample scarcity scenarios and significantly alleviates the distribution shift of support set and query set.

**Table 7:** Classification accuracy (%) under various configurations. Bold numbers indicate the best performance in each column.

Methods	Acc (4-way, 1-shot)	Acc (4-way, 5-shot)	Acc (2-way, 1-shot)	Acc (2-way, 5-shot)
Base	61.31	72.50	87.38	91.25
Base+tSF	61.81	75.93	87.63	91.75
Base+tSF+WFTBlock	62.31	78.31	88.50	92.13
Base+tSf+WFTBlock+BDcross	**69.63**	**79.13**	**90.75**	**94.15**

### Visual results and analysis

The t-Distributed Stochastic Neighbor Embedding (t-SNE) is a machine learning algorithm used for dimensionality reduction and visualization of high-dimensional data. It maps high-dimensional data into a low-dimensional space while preserving the local structural relationships among data points. We use ST-CFE to extract ECG high-dimensional features from different sets, and map them into the two-dimensional space through the t-SNE algorithm. The data points are color-coded according to their categories, providing an intuitive visualization of the distribution and clustering of each class in the feature space. For the 2-class classification task on ECG200, as shown in Fig 5(a). This indicates that the data maintains a certain structure in the high-dimensional space, and the two classes are largely separable, with only minor overlaps in a few regions. For the 5-class classification task on ECG5000, the t-SNE visualization in Fig 5(b) shows that most categories form relatively compact and non-overlapping clusters. The brown (PVC) and gray (R-on-T) have a slight inter-cluster mixing at the boundary, which is consistent with their known electrophysiological similarities. For the 7-class classification tasks on Chapman, as shown in Fig 5(c), the orange (SB) points form larger and more distinct clusters with clearer boundaries from other categories. This indicates that the ECG signals in this category have higher similarity in the feature space. In contrast, while the red (SI) and light blue (SVT) points are more scattered, suggesting greater feature diversity within these categories.

**Fig 5:**
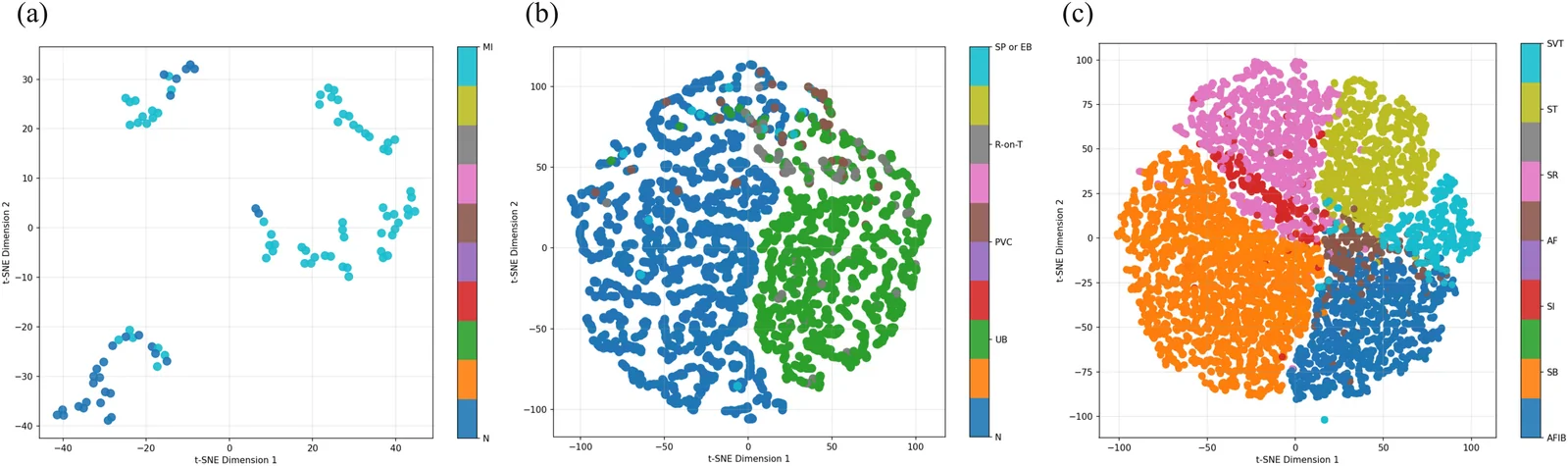
t-SNE feature maps on different sets. (a) ECG200. (b) ECG5000. (c) Chapman.

## Discussion

To address the issue of limited generalization ability in existing few-shot learning (FSL) methods, particularly in handling single feature representations and rare arrhythmia data in ECG classification tasks, this paper proposes a novel FSL method named STCP-Net. Compared with the existing FSL methods, the proposed model has significant advantages across multiple few-shot tasks (see Fig 6). The superior performance of the proposed method benefits from our two key contributions. First, the proposed improved prototypical network based on BDcross as an effective classifier alleviates the distribution deviation between support set and query set in FSL. Second, the ST-CFE module captures multi-angle ECG features such as time-frequency domain features and semantic information, thereby enriching the feature representation. Through the synergistic effect of the above two components, it achieves significant improvements in overall performance and generalization capability, especially for rare arrhythmia types.

**Fig 6:**
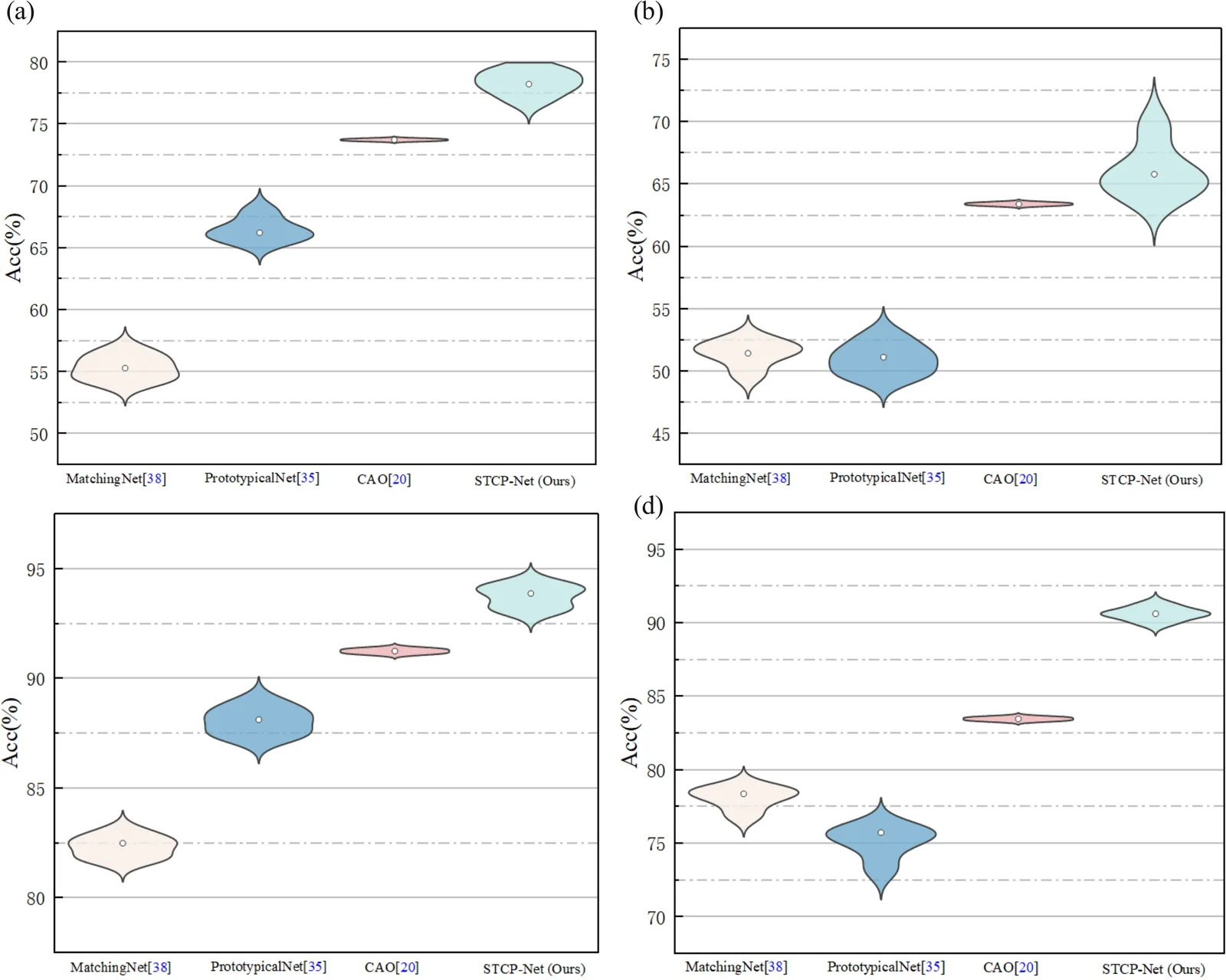
Performance comparison of different methods on Chapman. (a) 4-way 5-shot. (b) 4-way 1-shot. (c) 2-way 5-shot. (d) 2-way 1-shot.

Moreover, STCP-Net also shows low sensitivity to the size of meta-training data. Even when the size of the meta-training set is reduced to only 10%, its classification performance in the 2-way classification task remains close to that achieved with the full meta-training set (see Fig 7(a)). In order to further verify the technical advantages of ST-CFE, we evaluate the universality of ST-CFE module on Chapman’s full sample. Specifically, the dataset is divided into training and test at a ratio of 9:1. A standard supervised learning method is then employed for training and evaluation, and the results are compared with other existing methods. As shown in Fig 7(b), the comparison results demonstrate that ST-CFE indeed plays a positive role in performance improvement.

**Fig 7:**
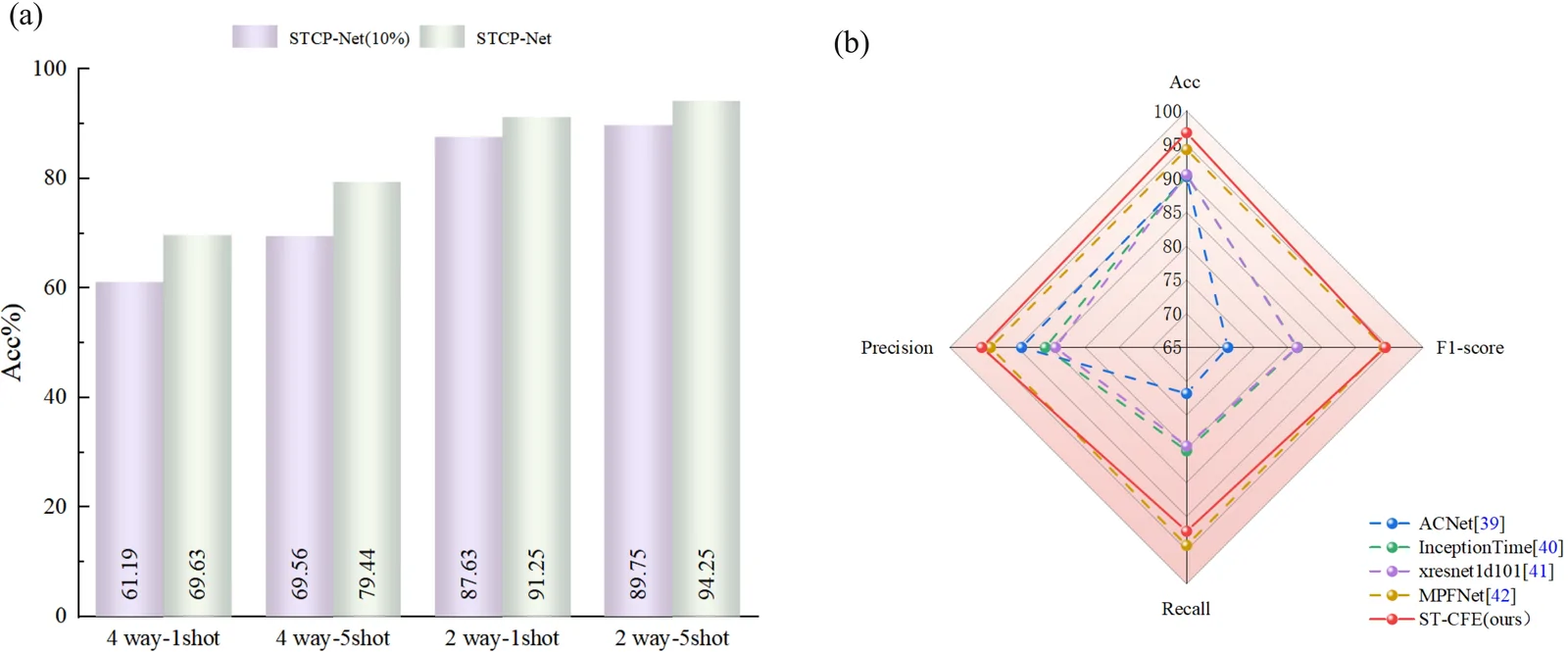
Comparison results on Chapman. (a) Comparison results of different tasks which meta-training set is reduced to 10%. (b) Radar images of different methods (ACNet [39], InceptionTime [40], xresnet1d101 [41], MPFNet [42]).

Although our study achieves competitive performance on the datasets used, we fully recognize several key limitations that need to be addressed in future work. First, we need to systematically verify the model’s generalization ability on more independent and heterogeneous public ECG databases. When training and test data come from different acquisition devices, patient populations, or signal qualities, domain shift can significantly degrade performance. To address this, we plan to conduct cross-dataset evaluations in future studies to better reflect real-world clinical applicability. Second, although STCP-Net is lightweight with only 83K trainable parameters and 193 MFLOPs per sample, it is still more complex than the original prototypical network (38K parameters, 57 MFLOPs). This increase comes from integrating multiple components. While STCP-Net improves performance, it also raises computational demands and parameter count, which could challenge deployment in resource constrained clinical settings. Therefore, our next step will focus on developing a more streamlined, efficient, and application-oriented simplified version. Finally, we note that the BDcross bias calibration mechanism may face stability issues in extremely low sample scenarios, such as the 1-shot settting. The mechanism estimates class prototypes and the bias ξ using support set statistics. In 1-shot settings, each class has only one sample, making these estimates highly sensitive to noise. Although current evaluations show the model retains some robustness under low data conditions, we will further investigate BDcross’s reliability in low data regimes to enable truly trustworthy few-shot clinical reasoning.

## Conclusion

In this paper, we propose a FSL method based on spectral-temporal convolution and an improved prototypical network. This proposed method extracts key features through the ST-CFE module and uses the improved prototypical network for classification. In ST-CFE, we use WFTBlock to extract the global spectrum and local time-frequency features of the signal, combining SBSP to capture the overall semantic information. This design enables the model to focus more accurately on key features. In addition, in order to reduce the distribution gap between the support set and the query set, we introduce BDcross to improve the prototypical network, which significantly enhances the adaptability of STCP-Net to rare categories. The experimental results show that STCP-Net achieves higher accuracy and stronger generalization capability than existing few-shot learning methods in ECG classification tasks, fully validating the effectiveness and superiority of the proposed method.
